# Association of cerebellar and pre-motor cortex gray matter density with subjective intoxication and subjective response following acute alcohol intake

**DOI:** 10.1038/s41598-023-34546-5

**Published:** 2023-05-05

**Authors:** Bethany A. Stennett-Blackmon, Landrew Sevel, Jeff Boissoneault

**Affiliations:** 1 Department of Clinical and Health Psychology, University of Florida, P.O. Box 100165, Gainesville, FL 32610 USA; 2 Center for Pain Research and Behavioral Health, University of Florida, Gainesville, FL USA; 3 Osher Center for Integrative Health, Vanderbilt University Medical Center, Nashville, TN USA; 4 Department of Physical Medicine and Rehabilitation, Vanderbilt University Medical Center, Nashville, TN USA

**Keywords:** Human behaviour, Predictive markers, Neuroscience

## Abstract

Acute alcohol intake produces subjective intoxication (SI) and response (SR; e.g., valanced stimulation and sedation), which has important implications for alcohol-related risk. Individuals who experience less SI may be more likely to engage in risky behaviors while drinking. Gray matter morphometry in brain regions underlying cognitive and affective processes may help to inform individual differences in subjective intoxication and response. The subjective effects of alcohol vary between limbs of the blood alcohol concentration (BAC) curve (i.e., whether BAC is rising or falling; acute tolerance). We examined the relationship between gray matter density (GMD) and SI/SR as a function of BAC limb. Healthy social drinkers (N = 89; 55 women) completed an alcohol challenge paradigm (target BAC = 0.08 g/dL) and structural magnetic resonance imaging (MRI). Participants completed measures of SR and SI on ascending and descending BAC limbs. Association between GMD and SI/SR on each limb were assessed using whole-brain, voxel-wise general linear models. GMD estimates were extracted from significant clusters. Differences in association of GMD and SI/SR between limbs were assessed using hierarchical regression. Significant associations of SI with GMD on the ascending limb were observed in the cerebellum. A significant association between SR and GMD on the descending limb were observed in the pre-motor cortex (BA6) and cerebellum. We identified common and unique associations among cerebellum and pre-central gyrus structures with SI and SR between BAC limbs. Functional imaging studies may further clarify unique dimensions of subjective alcohol effects linked to the observed structural associations.

## Introduction

Subjective intoxication (SI) and response (SR) are aspects of the self-reported experience of alcohol effects, including stimulation and sedation (we use SR to reference experiences specifically measured by the Subjective Effects of Alcohol Scale)^1,2^. Evidence indicates that perception of intoxication may strongly influence the likelihood of engaging in hazardous behaviors after drinking, including driving^3^. Individuals who experience lower SI at a given blood alcohol concentration (BAC) may feel less impaired and be more willing to engage in risky behaviors. Understanding intraindividual variability in subjective experience will help identify individuals at neurobehavioral risk from alcohol consumption and inform intervention efforts.

Several biopsychosocial factors contribute to individual differences in SI/SR. Alcohol pharmacokinetics influences SI, with more rapid increases in BAC producing greater SI^4^. These factors also vary within a drinking episode as individuals tend to report greater intoxication at a given BAC on the ascending than descending limb of the BAC curve (i.e., acute tolerance)^5^. Although SI/SR and some aspects of behavioral impairment trend lower on the descending limb, many aspects of behavioral function are equally impaired. For example, individuals report higher willingness to drive on the descending limb despite equivalent behavioral impairment (Amlung et al., 2014; Marczinski & Fillmore, 2009)^6,7^. Additionally, individuals engaging in regular heavy drinking (HDI) experience functional tolerance to SI, resulting in lower SI than moderate or light drinkers^8^. HDI may have lower gray matter density (GMD) than moderate drinkers in multiple brain areas involved in attention, motivation, and reward (e.g., prefrontal cortex, anterior and posterior cingulate cortex, striatum, and insula; Xiao et al., 2015)^9^. These changes progress over time in heavy drinkers^10^, implying alcohol use results in structural changes in regions underlying SI. Although these data suggest important relationships between GMD in these regions and individual differences in SI and SR, this possibility has not been examined in low-risk drinkers.

In this study, we examined the association between SI/SR and GMD following acute alcohol administration in healthy, social drinkers across the ascending and descending limbs of the BAC curve. Given evidence that HDI have both a) reduced SI/SR in response to alcohol use; and b) lower GMD in brain regions underlying higher cognitive functions, we predicted that social drinkers who report lower SI/SR would exhibit lower GMD in these areas, and that these associations would *not* be completely captured by the non-specific effects of alcohol intake (i.e., placebo).

## Methods

### Study design

The present investigation was part of a larger study of behavioral and neural processes of alcohol analgesia (NCT04925076). Relevant methods are presented below. All participants provided informed consent prior to data collection and all research was performed in accordance with relevant guidelines and regulations.

### Participants

Healthy social drinkers (N = 89; 55 women) completed the following questionnaires during an initial screening session: the Alcohol Use Disorders Identification Test (AUDIT)^11^; Alcohol Use Questionnaire (AUQ)^12^; Beck Depression Inventory-II (BDI-II)^13^; demographic, medical histories, and family history of alcohol problems. Individuals were excluded for: consuming < 1 alcohol containing beverage per month during the 6 months prior to assessment, alcohol naïveté, history of substance or alcohol use disorder, lifetime substance dependence, problematic alcohol use (AUDIT ≥ 8); present psychotic disorder or significant depression (BDI-II > 20), under-controlled metabolic disease, serious medical illness or neurological disease, and current nicotine use.

### Alcohol administration

Participants were administered alcohol and placebo beverages (target breath alcohol concentration [BrAC] 0.08 g/dL and 0.00 g/dL, respectively) during counterbalanced, double-blind sessions separated by at least 48 h. Participants fasted for 4 h and abstained from alcohol for 24 h prior to administration^14,15^. With the exception of analgesics or allergy medications, normal daily medication use was allowed. Positive baseline BrAC assessment, urine drug screen (benzodiazepine, cocaine, methamphetamine, morphine, tetrahydrocannabinol) or pregnancy tests were exclusionary. One hour prior to beverage administration, participants consumed a ~ 220 kcal breakfast. The amount of 95% medical-grade ethanol needed to achieve BAC of 0.08 g/dL^16^ was mixed in a 1:3 ratio with sugar-free lemon-lime soda. Placebo beverages consisted only of soda and a small amount of alcohol was placed on the rim of the glass and surface of the drink in order to mask the study condition. Beverages were consumed in two servings within 5 min, after which participants rinsed their mouths with water^17,18^. At the end of each study session, participants were asked if they believe they received an alcoholic beverage in order to assess placebo effectiveness.

### Alcohol concentration assessment

A breathalyzer (CMI, Inc., Owensboro, KY, USA) was used to assess BrAC before and after the MRI scan. During scanning, QED A150 tests (OraSure Technologies, Inc., Bethlehem, PA, USA) assessed salivary alcohol concentration (SAC)^19^. BrAC and SAC samples were collected in tandem 10- and 20-min following beverage consumption. Participants were then placed in the scanner and SAC samples were collected three times during scanning between acquisitions. The total amount of time in the scanner was approximately 50 min. BrAC and SAC were sampled again immediately following the MRI scan. BrAC was monitored and participants were transported home when BrAC reached ≤ 0.02 g/dL^20^.

### Subjective intoxication and response assessment

Fifteen minutes post-administration (ascending limb), between the 10 and 20 min post-beverage administration BrAC and SAC sample collections, participants rated subjective intoxication (SI) with a 100 mm VAS (“Not at all intoxicated” to “Most intoxicated imaginable”). Immediately following the post-scan BrAC and SAC sample collection (descending limb), participants completed the same VAS.

Subjective Response (SR) was assessed at the same points as SI using the Subjective Effects of Alcohol Scale (SEAS; Morean et al., 2013; as noted above, SR is used to refer to aspects of the subjective alcohol experience as measured by the SEAS throughout the manuscript)^2^. The SEAS measures stimulating and sedating effects of alcohol (i.e., “high” vs. “low” arousal) of both positive and negative valence. Individuals rate the extent they are experiencing fourteen feelings on 0–10 numeric rating scales. Example items for each combination of arousal and valence include “lively” and “fun” (High Positive; HP), “rude” and “aggressive” (High Negative; HN), “calm” and “mellow” (Low Positive; LP), and “dizzy” and “woozy” (Low Negative, LN). The SEAS shows internal consistency across subscales (0.80 < α < 0.94), good convergent and discriminant validity compared with other measures of subjective alcohol response, and measurement invariance across BAC limbs^2^.

### Image acquisition

Participants were positioned in a Siemens Prisma 3 T scanner (64-channel head coil) 25–30 min post-beverage administration, in which a three-dimensional T1-weighted MP-RAGE whole-brain structural image was acquired. Parameters were FOV = 256 mm, voxel-wise resolution = 0.8 × 0.8 × 0.8 mm, 320 contiguous sagittal slices, acceleration factor = 2, and TR/TE/FA = 2000 ms/2.99 ms/8°.

### Voxel-based morphometry (VBM) analyses

VBM analyses were conducted in SPM12 (Welcome Trust Centre for Neuroimaging, University College London, London, UK) using standard, previously described methods^21^. Briefly, steps included (1) manual reorientation of T1 images; (2) down-sampling to 1.5 mm^3^ resolution; (3) segmentation in native space and generation of a study-specific group template using DARTEL^22^; (4) alignment of individual images to the group template; (5) normalization of the group template and then individual scans to MNI space); and (6) smoothing with an 8 mm^3^ Gaussian kernel.

### Statistical analysis

Paired-sample t-tests were performed to assess differences between limb and condition in SI. The subscales of the SEAS were entered into a repeated measures ANOVA to examine these differences as well.

To assess associations among SI and SR and gray matter density (GMD), voxel-wise multiple regression was performed. Unique regression models were performed, where the independent variable of interest was one of each dimension of SI/SR, including the single item SI measure, the four subscales of the SEAS (i.e., HP, HN, LP, and LN) and whole brain, voxel-wise GMD as the dependent variable. The same procedure was completed for subject reports on the ascending and descending limbs. In all models, age, sex, and total intracranial volume (TIV) were entered as covariates. Sex differences in GMD are well documented and potential associations between sex and SI/SR may exist. Correlations between sex, SI/SR, and where relevant SI/SR by GMD interactions were performed. Results were assessed under cluster size-based family-wise error (FWE) correction (*p*_height_ < 0.001, uncorrected; *p*_cluster_ < 0.05, FWE). Regions were labelled using the AAL3 atlas^23^. Mean GMD within significant clusters was extracted.

To discern whether results were limb- (ascending vs. descending) and condition-specific (alcohol vs. placebo), separate hierarchical regressions were performed using IBM SPSSv27. For SI and SR measures found to predict GMD, ratings from the placebo condition were regressed on those from the alcohol condition. Unstandardized residuals were saved to generate a variable that measures the substance-specific effects of alcohol. In subsequent regressions, mean GMD estimates for each significant cluster were entered as dependent variables. In the first step, ratings from the placebo condition were entered, along with covariates (TIV, sex, and age). In the second step, the alcohol-specific orthogonalized ratings were entered, with significant changes in variance explained in step two indicating a unique association above and beyond placebo. The same procedure was performed but with SI/SR ratings from the limb opposite to that found in voxel-wise analyses. The absence of significant associations between the orthogonalized variable and GMD suggested limb-specific effects.

### Informed consent

The study was reviewed and approved by the University of Florida Institutional Review Board. All participants provided written informed consent.

## Results

### Demographics and alcohol use

Participants (*N* = 89) averaged 26.20 (*SD* = 4.44) years of age. 79.8% identified as White, 3.4% as Black/African American, 12.4% as Asian, 4.5% as another race/multiple races and 21.6% were Hispanic/Latinx. Typical daily alcohol intake over the last 6 months averaged 0.46 oz. of absolute ethanol (*SD* = 0.29), less than one standard drink. AUDIT scores indicated low alcohol use disorder symptomatology (*M* = 4.67, *SD* = 1.59). The proportion of participants reporting a biological parent with past or current alcohol use problems was 24.72% [n = 22; (women: n = 15), (men: n = 7)]. Family history was not found to correlate with any BrAC or subjective ratings in our study and as such was not entered as a covarite in GMD analyses. Information collected during the screening session indicated 40.5% (n = 36) of participants reported using at least one daily medication. Of these, the most common were hormonal birth control (47%, n = 17), antidepressants (25%, n = 9), and allergy medications (22%, n = 8).

### BrAC/SAC and Placebo effectiveness

**Figure 1:**
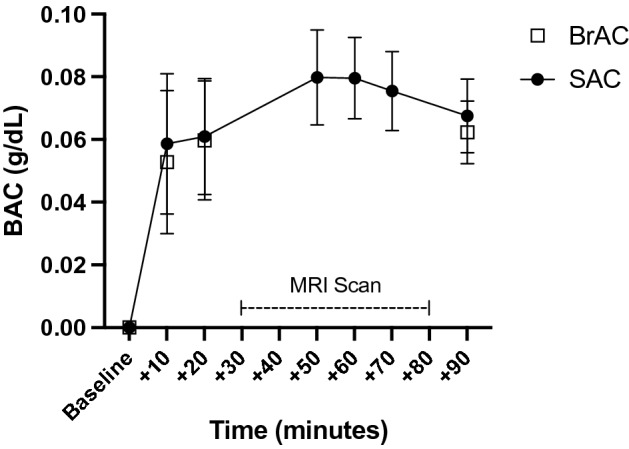
BrAC and SAC measurements. BrAC and SAC measurements plotted over time. BrAC measurements were collected before and after the MRI scan. SAC measurements were collected pre-, during, and post-scan.

Participant BrAC averaged 0.053 g/dL (*SD* = 0.02 g/dL; Range: 0.014–0.118) 10 min post-drink administration and 0.060 g/dL (*SD* = 0.02; Range: 0.021–0.110) 20 min post-drink. BrAC, SAC, SEAS, and SI measures were collected after MRI scanning was complete to assess the descending limb (*M* = 87 min, *SD* = 10 min post-drink). Average descending limb BrAC was 0.062 g/dL (*SD* = 0.01; Range: 0.043–0.087). BrAC significantly differed between assessment points on the ascending (taken as the average of the 10 min and 20 min measures) and descending limbs (*t*(88) = 2.66, *p* = 0.01, d = 0.39). Saliva alcohol concentration (SAC) samples were collected in tandem with BrAC measurements for the ascending [10 min post-drink (*M* = 0.059; *SD* = 0.02; Range: 0.01–0.12); 20 min post-drink (*M* = 0.060; *SD* = 0.02; Range: 0.02–0.10)] and descending limb [post-scan (*M* = 0.068; *SD* = 0.01; Range: 0.04–0.10)]. SAC samples collected during MRI acquisition were as follows: Sample 1 (*M* = 0.080; *SD* = 0.02; Range: 0.04–0.12), Sample 2 (*M* = 0.080; *SD* = 0.01; Range: 0.05–0.12), Sample 3 (*M* = 0.075; *SD* = 0.01; Range: 0.05–0.12). BrAC and SAC curves over time are shown in Fig. 1.

In the alcohol session, 100% of participants reported they believed the drink they consumed contained alcohol. In the placebo session, 46% of participants endorsed belief that their beverage contained alcohol.

### Subjective intoxication and response (SEAS)

Consistent with acute tolerance, ratings of SI in the alcohol condition averaged 37.96 (*SD* = 20.19) on the ascending limb and 29.76 (*SD* = 18.75) on the descending limb, which significantly differed (*t*(88) = 3.59, *p* = 0.0005, d = 0.42). For placebo sessions, ratings of the ascending limb (*M* = 9.01, *SD* = 9.87) and descending limb (*M* = 5.23, *SD* = 7.90) also significantly differed (*t*(88) = 3.48, *p* = 0.0008, d = 0.42; Supplemental Fig. 1).

Repeated measures ANOVAs were performed on SEAS ratings. There was a significant effect of condition on LN (M_alc_ = 7.75 vs M_plac_ = 2.07; (F(1, 352) = 83.47, *p* < 0.0001; η^2^ = 0.192), HP (M_alc_ = 22.38 vs M_plac_ = 18.17; (F(1, 352) = 18.54, *p* < 0.0001; η^2^ = 0.049), and HN (M_alc_ = 0.787 vs M_plac_ = 0.225; (F(1, 352) = 10.01, *p* = 0.0017; η^2^ = 0.027) subscales, but not LP (*p* = 0.83). There was a significant effect of limb on HP (M_asc_ = 21.88 vs M_desc_ = 18.67; (F(1, 352) = 10.78, *p* = 0.001; η^2^ = 0.028) and HN (M_asc_ = 0.697 vs M_desc_ = 0.315; (F(1, 352) = 4.63, *p* = 0.032; η^2^ = 0.013) subscales, but not LP or LN (*p* > 0.47). No limb x condition interactions were noted. SI and SEAS ratings are shown in Table 1.

**Table 1:** Subjective Intoxication and SEAS Ratings.

	Alcohol session		Placebo session	
	Ascending	Descending	Ascending	Descending
	M (SD)	M (SD)	M (SD)	M (SD)
Subjective intoxication	37.96 (20.19)	29.76 (18.75)	9.01 (9.87)	5.23 (7.90)
Low positive	29.54 (7.24)	30.19 (7.75)	29.79 (7.18)	30.28 (7.57)
Low negative	7.55 (7.37)	7.96 (7.46)	2.01 (3.50)	2.14 (3.92)
High positive	24.39 (9.28)	20.37 (9.46)	19.37 (8.81)	16.97 (9.33)
High negative	1.08 (2.66)	0.49 (1.38)	0.31 (1.31)	0.14 (0.71)

### Subjective intoxication and GMD associations

As noted above, sex differences may influence alcohol experience. However, in our data set, sex was only correlated with one facet of subjective response, SEAS high positive ratings on the descending limb (r = 0.31, *p* = 0.004). However, assessment of sex by rating interactions on GMD revealed no significant effects, as such, it was not included as a covariate of interest in voxel-wise regressions.

**Figure 2:**
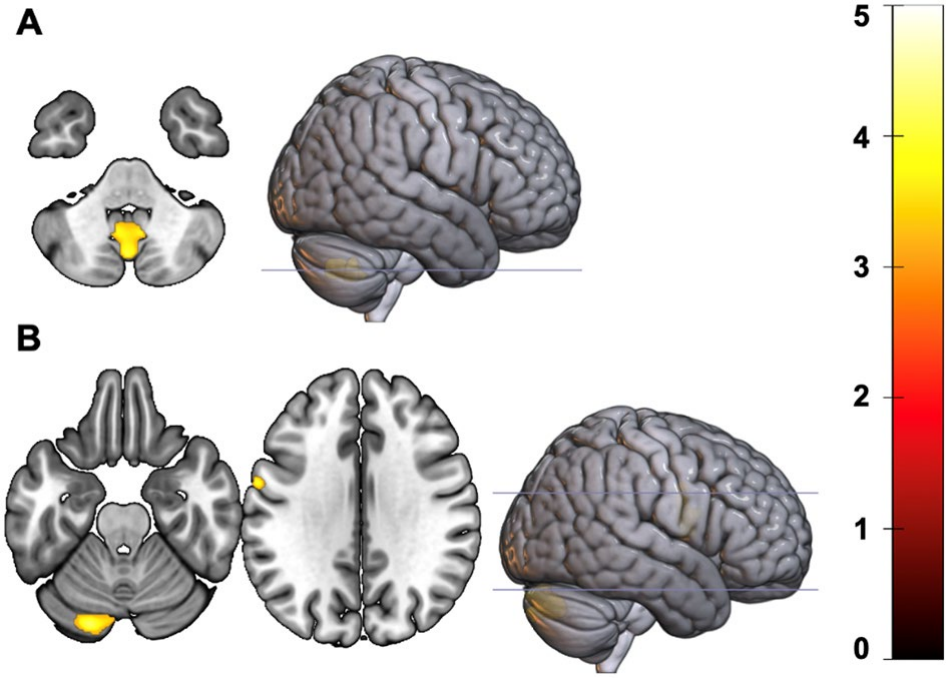
Axial sections and anterior rendering of regions in which gray matter density was associated with subjective intoxication and response. (**A**) displays regions significantly associated with subjective intoxication on the ascending limb while (**B**) displays associations with descending limb, Low Positive SEAS ratings.

Significant associations between GMD and SI were identified within one cluster of the cerebellum wherein greater SI on the ascending limb was associated with greater GMD (Table 2, Fig. 2A). No significant associations were found between ratings of SI and GMD on the descending limb.

**Table 2:** Regions in which GMD Is Predicted by Ascending BrAC Subjective Intoxication.

Region	Coordinates			k	% Cluster	Peak *t*
	X	Y	Z			
Vermis 8	− 2	− 71	− 42	724	23.20	4.15
L Cerebellum 9					21.96	
Vermis 9					18.09	
L Cerebellum 8					16.71	
R Cerebellum 9					9.25	
Unlabeled					8.29	
R Cerebellum 8					1.80	
L Cerebellum 7b					0.55	
L Cerebellum Crus 2					0.14

### SEAS and GMD associations

Significant associations between SEAS subscales and GMD were identified for LP SEAS ratings on the descending limb within two clusters, one in the cerebellum and another spanning aspects of the pre/postcentral and temporal gyrus, wherein greater LP ratings were associated with greater GMD (Table 3, Fig. 2B). No significant associations between the other scales of the SEAS on either limb were identified.

**Table 3:** Regions in which GMD Is Predicted by Descending BrAC Low Positive SEAS Ratings.

Region	Coordinates			k	% Cluster	Peak *t*
	X	Y	Z			
L Cerebellum Crus 1	− 20	− 83	− 24	1374	55.82	5.12
L Cerebellum Crus 2					40.39	
Unlabeled					1.82	
L Cerebellum 7b					0.80	
L Cerebellum 6					0.66	
L Cerebellum 8					0.51	
L Precentral	− 60	− 2	32	460	40.00	4.04
L Postcentral					33.04	
L Rolandic Operculum					12.61	
L Frontal Inferior Operculum					10.87	
Unlabeled					3.48	

### Alcohol specific and non-specific associations with GMD

Separate hierarchical regressions were performed to assess the associations of alcohol intoxication specific and non-specific effects on each of the three GMD clusters identified above as significant in voxel-wise analyses (See Supplemental Tables 1–3).

For the cerebellar cluster where SI ratings on the ascending limb significantly predicted GMD, significant variance in GMD was explained by the initial model including placebo condition scores and covariates [F(4, 84)5.089, *p* < 0.001, R^2^ = 0.195). However, scores from the placebo condition were not individually predictive of GMD (β = 0.126, *p* > 0.05). Adding alcohol-specific SI scores significantly increased the variance explained by the model (FΔ(1, 83) = 13.059, *p* < 0.001, R^2^Δ = 0.109) and were individually predictive of GMD (β = 0.333, *p* > 0.001).

Regression analyses for the cerebellar cluster (LP ratings on the descending limb significantly predicted GMD) indicated significant variance in GMD was explained by the initial model including placebo condition scores and covariates [F(4, 84) = 8.993, *p* < 0.001, R^2^ = 0.300), with placebo LP ratings individually predicting cerebellar GMD (β = 0.235, *p* < 0.001). Adding alcohol-specific SI scores significantly increased the variance explained by the model (FΔ(1, 83) = 6.716, *p* < 0.01, R^2^Δ = 0.052). In this new model, both alcohol (β = 0.229, *p* > 0.01) and placebo (β = 0.325, *p* > 0.001) scores individually predicted GMD.

For the precentral gyrus cluster significantly associated with LP SEAS ratings on the descending limb, significant variance in GMD was explained by the initial model [F(4, 84) = 14.332, *p* < 0.001, R^2^ = 0.406). Placebo condition scores individually predicted GMD values (β = 0.188, *p* < 0.05). The addition of alcohol-specific ratings significantly increased the variance explained by the model (FΔ(1, 83) = 14.722, *p* < 0.001, R^2^Δ = 0.090). Both alcohol and placebo scores significantly predicted GMD (β = 0.300, *p* > 0.001; and β = 0.187, *p* > 0.05, respectively).

### Limb specific associations with GMD

Additional hierarchical regressions were performed to determine whether the observed associations between each of the three GMD clusters identified above and SI/SR were unique to the limb in which the association was initially identified. For example, voxel-wise regression identified a significant association between SI and cerebellar GMD in the ascending limb. As such, ratings from the *descending* limb were entered into a regression model. For these analyses, and the parallel analyses with opposite limb measures of the SEAS, no significant association was found between placebo or alcohol condition ratings and GMD, and significant associations were found only between covariates and outcome variables (Supplemental Tables 4–6). Thus, associations between SI and cerebellar GMD appeared to be specific to the ascending limb, while associations between LP SEAS ratings and cerebellar and pre-/post-central gyrus GMD appeared to be specific to the descending limb.

## Discussion

This study assessed relationships between subjective response and GMD following alcohol administration in social drinkers on both the ascending and descending limbs of the BAC curve. Results indicated greater ascending limb subjective intoxication (SI) was associated with greater cerebellar GMD and with (LP) descending limb SEAS ratings in the cerebellum and left primary motor and somatosensory cortices. These associations appeared to be unique to the BrAC limb on which they were detected and from the non-specific effects of beverage administration (i.e., placebo). These results are consistent with hypotheses that lower SI and attenuated subjective response would be linked with lower GMD, a similar association as that found in HDI.

Given SI is a multidimensional construct^1,24^ subsumed by multiple neural systems, it is notable that we found SI on the ascending limb was associated with GMD only within the cerebellum. Ratings of the positive sedating effects of alcohol intake (e.g., relaxation, mellowness) were also associated with cerebellar GMD on the descending limb. Growing evidence implicates the cerebellum in diverse neurocognitive and behavioral functions given its strong interconnectedness with numerous brain regions^25^. A role for SI in predicting cerebellar gray matter is consistent with prior research showing regional decreases in cerebellar blood flow^26^ and glucose metabolism^27^ following alcohol doses similar to that in this study. The cerebellum also underlies alcohol-induced disruption of motor coordination^28^, eye movement^29^, cognitive control^30^, and complex integrated behaviors (i.e., driving)^31,32^. Alcohol-induced metabolic changes in the cerebellum (i.e., greater acetate and lower glucose utilization) have been associated with SI^27^, and low level of response to alcohol has been linked to greater cerebellar activation during working memory task performance in adolescents^33^. Thus, it is possiblethat cerebellar structure may be a useful indicator of multiple aspects of subjective response to alcohol across limbs.

While acute tolerance to alcohol is a well-established phenomenon, the most consistent indicators of its effects pertain to subjective response to alcohol^34,35^. Our results provide evidence of structural correlates of subjective response, which may be distinct between the BAC limbs. Although SI predicted cerebellar GMD on the ascending limb, GMD in clusters encompassing left primary somatosensory and motor cortices and cerebellum were significantly associated with the LP subjective response only on the descending limb. Both the cerebellum and the primary somatosensory and motor cortices are critical structures underlying voluntary motor activity. It seems intuitive that GMD in these regions may be related to subjective response given alcohol’s psychomotor effects. There are well-documented connections between the motor cortices and cerebellum, which play an important role in coordination and error monitoring via pathways involving primary somatosensory and motor cortex, cerebellum, and thalamus^36^. Individual differences in these structures and related cortico-cerebellar networks may play an important role in acute alcohol response, including both intoxication and positive/pleasant aspects of sedation.

It is conceivable that individual differences in brain structure such as those identified here may indicate risk for increased alcohol consumption due to their association with blunted subjective response after drinking. Evidence suggests HDI report lower SI ratings than social drinkers in response to acute alcohol challenge^8^. Furthermore, AUD and chronic heavy alcohol intake are associated with decreased GM volume. Our data suggest that these processes may exist on a continuum, with higher ratings of SI being associated with greater GMD, and potentially protective against response blunting, and development of AUD or dependence.

### Limitations, future directions

To our knowledge, there are no previous investigations of brain structure and subjective response to acute alcohol intoxication. Our results provide a novel contribution to elucidation of neural factors in alcohol response and individual differences. However, some limitations must be considered to fully contextualize this work. Our sample was not representative of certain demographic groups and age ranges in which experiences of SI may meaningfully vary. Likewise, our results are unable to speak to associations in those with AUD, though we suspect similar regional involvement. Future studies inclusive of these factors and groups may meaningfully expand upon these results. Also, the study paradigm was designed to limit BAC variability between participants; interindividual differences in BrAC may uniquely relate to GMD. Future studies designed to assess interindividual differences in interactions between GMD and subjective experience at variable BrAC levels are recommended. Our study found novel associations between descending limb LP ratings of the SEAS and GMD while there were no significant differences in LP between limbs or conditions. It is possible that this association in interindividual differences in GMD and limb/condition-specific LP ratings exists despite the absence of group-level differences. Other sources may also account for this finding such as the relatively high LP ratings across limbs and conditions, a potential ceiling effect possibly related to the relaxed experimental setting. Assessment of these relationships in varied experimental or naturalistic contexts are needed to clarify this finding.

Use of certain daily medications was permitted in this study, though medications taken on the day of scanning were not assessed. It is possible that certain medications could influence an individual’s experience of alcohol intoxication. Although we did not find associations between family history of alcohol problems and subjective report or BrAC, it is well known that these factors can influence alcohol experience. Only a small portion of our sample reported family history of alcohol use problems and subsequent studies with more balanced samples may be more appropriately powered to find such associations. Finally, our results are limited to features of brain structure that may correspond with acute tolerance to alcohol. However, they do identify promising targets for future investigations such as structural/functional connectivity, or signal variability and complexity, that may be involved in this phenomenon.

## Conclusions

This study identified significant associations between GMD and interindividual differences in subjective alcohol response. We identified common and unique associations among the cerebellum and primary somatosensory and motor cortices as apredicted by SI and Low Positive aspects of alcohol response between BAC limbs. These structures should be considered when evaluating structural predictors for alcohol response and alcohol-related risks. Multi-modal and functional imaging studies may further clarify unique dimensions of subjective response linked to the observed structural associations.

## Supplementary Information

Supplementary Information.

## Data Availability

The datasets generated during and/or analyzed during the current study are available from the corresponding author on reasonable request.
