# Supplementary material for: Association of cerebellar and pre-motor cortex gray matter density with subjective intoxication and subjective response following acute alcohol intake
Source: Sci Rep. 2023 May 5;13:7340. doi: 10.1038/s41598-023-34546-5 (PMC10163021; doi:10.1038/s41598-023-34546-5)
Supplement: Supplementary file 1 — Supplementary Information. [file 41598_2023_34546_MOESM1_ESM.docx]

**Supplemental Materials**

**Supplemental Figure 1. Subjective Intoxication Ratings.** Ratings of Subjective intoxication significantly differed between ascending and descending limbs of the BAC curve in both Alcohol and Placebo conditions.

| Supplemental Table 1. Hierarchical Regression Results for Ascending Limb Subjective Intoxication Ratings and Cerebellar GMD | | | | | | | | | |
| --- | --- | --- | --- | --- | --- | --- | --- | --- | --- |
| Variable | B | β | SE | *t* | F | F Δ | *R*^2^ | *R*^2^ Δ |  |
| Model 1 |  |  |  |  | 5.089*** | - | .195 | - |  |
| TIV | .000 | .204 | .000 | 1.548 |  |  |  |  |  |
| Sex | .000 | .266 | .024 | 2.018* |  |  |  |  |  |
| Age | -.002 | -.092 | .002 | -.912 |  |  |  |  |  |
| Placebo | .001 | .126 | .001 | 1.251 |  |  |  |  |  |
| Model 2 |  |  |  |  | 7.268*** | 13.059*** | .305 | .109 |  |
| TIV | .000 | .235 | .000 | 1.898 |  |  |  |  |  |
| Sex | .042 | .228 | .023 | 1.841 |  |  |  |  |  |
| Age | -.002 | -.108 | .002 | -1.114 |  |  |  |  |  |
| Placebo | .001 | .132 | .001 | 1.400 |  |  |  |  |  |
| Alcohol | .002 | .333 | .000 | 3.614*** |  |  |  |  |  |
| Notes: *p<0.05, **<0.01, ***<0.001 | | | | | | | | | |

| Supplemental Table 2. Hierarchical Regression Results for Descending Limb Low Positive SEAS Ratings and Cerebellar GMD | | | | | | | | | |
| --- | --- | --- | --- | --- | --- | --- | --- | --- | --- |
| Variable | B | β | SE | *t* | F | F Δ | *R*^2^ | *R*^2^ Δ |  |
| Model 1 |  |  |  |  | 8.993*** | - | .300 | - |  |
| TIV | .000 | .450 | .000 | 3.648*** |  |  |  |  |  |
| Sex | .000 | .001 | .023 | .012 |  |  |  |  |  |
| Age | -.002 | -.076 | .002 | -.828 |  |  |  |  |  |
| Placebo | .004 | .235 | .001 | 3.535*** |  |  |  |  |  |
| Model 2 |  |  |  |  | 9.027*** | 6.716** | .352 | .052 |  |
| TIV | .000 | .441 | .000 | 3.689*** |  |  |  |  |  |
| Sex | .001 | .005 | .022 | .040 |  |  |  |  |  |
| Age | -.001 | -.063 | .002 | -.708 |  |  |  |  |  |
| Placebo | .004 | .325 | .001 | 3.646*** |  |  |  |  |  |
| Alcohol | .003 | .229 | .001 | 2.591** |  |  |  |  |  |
| Notes: *p<0.05, **<0.01, ***<0.001 | | | | | | | | | |

| Supplemental Table 3. Hierarchical Regression Results for Descending Limb Low Positive SEAS Ratings and Precentral Gyrus GMD | | | | | | | | | |
| --- | --- | --- | --- | --- | --- | --- | --- | --- | --- |
| Variable | B | β | SE | *t* | F | F Δ | *R*^2^ | *R*^2^ Δ |  |
| Model 1 |  |  |  |  | 14.332*** | - | .406 | - |  |
| TIV | .000 | .591 | .000 | 5.200*** |  |  |  |  |  |
| Sex | .000 | .000 | .016 | .000 |  |  |  |  |  |
| Age | -.003 | -.170 | .001 | -2.001* |  |  |  |  |  |
| Placebo | .002 | .188 | .001 | 2.214* |  |  |  |  |  |
| Model 2 |  |  |  |  | 16.283*** | 14.722*** | .495 | 0.90 |  |
| TIV | .000 | .579 | .000 | 5.490*** |  |  |  |  |  |
| Sex | .001 | .004 | 0.15 | .041 |  |  |  |  |  |
| Age | -.002 | -.153 | .001 | -1.937 |  |  |  |  |  |
| Placebo | .002 | .187 | .001 | 2.378* |  |  |  |  |  |
| Alcohol | .003 | .300 | .001 | 3.837*** |  |  |  |  |  |
| Notes: *p<0.05, **<0.01, ***<0.001 | | | | | | | | | |

| Supplemental Table 4. Opposing Limb Hierarchical Regression Results for Descending Limb Subjective Intoxication Ratings and Cerebellar GMD | | | | | | | | | |
| --- | --- | --- | --- | --- | --- | --- | --- | --- | --- |
| Variable | B | β | SE | *t* | F | F Δ | *R*^2^ | *R*^2^ Δ |  |
| Model 1 |  |  |  |  | 5.433*** | - | .206 | - |  |
| TIV | .000 | .126 | .000 | .931 |  |  |  |  |  |
| Sex | .056 | .303 | .024 | 2.295* |  |  |  |  |  |
| Age | -.002 | -.095 | .002 | -.954 |  |  |  |  |  |
| Placebo | .002 | .169 | .001 | 1.651 |  |  |  |  |  |
| Model 2 |  |  |  |  | 4.308** | .022 | .206 | .000 |  |
| TIV | .000 | .125 | .000 | .913 |  |  |  |  |  |
| Sex | .056 | .303 | .000 | 2.278* |  |  |  |  |  |
| Age | -.002 | -.094 | .002 | -.941 |  |  |  |  |  |
| Placebo | .002 | .169 | .001 | 1.644 |  |  |  |  |  |
| Alcohol | .000 | .014 | .001 | .147 |  |  |  |  |  |
| Notes: *p<0.05, **<0.01, ***<0.001 | | | | | | | | | |

| Supplemental Table 5. Opposing Limb Hierarchical Regression Results for Ascending Limb Low Positive SEAS Ratings and Cerebellar GMD | | | | | | | | |
| --- | --- | --- | --- | --- | --- | --- | --- | --- |
| Variable | B | β | SE | *t* | F | F Δ | *R*^2^ | *R*^2^ Δ |
| Model 1 |  |  |  |  | 8.993*** | - | .300 | - |
| TIV | .000 | .450 | .000 | 3,648*** |  |  |  |  |
| Sex | .000 | .001 | .023 | .012 |  |  |  |  |
| Age | -.002 | -.076 | .002 | -.828 |  |  |  |  |
| Placebo | .004 | .325 | .001 | 3.535*** |  |  |  |  |
| Model 2 |  |  |  |  | 7.122*** | .048 | .300 | .000 |
| TIV | .000 | .451 | .000 | 3.631*** |  |  |  |  |
| Sex | .000 | .001 | .023 | .009 |  |  |  |  |
| Age | -.002 | -.073 | .002 | -.776 |  |  |  |  |
| Placebo | .004 | .325 | .001 | 3.515*** |  |  |  |  |
| Alcohol | .000 | -.020 | .001 | -.218 |  |  |  |  |
| Notes: *p<0.05, **<0.01, ***<0.001 | | | | | | | | |

| Supplemental Table 6. Opposing Limb Hierarchical Regression Results for Ascending Limb Low Positive SEAS Ratings and Precentral Gyrus GMD | | | | | | | | |
| --- | --- | --- | --- | --- | --- | --- | --- | --- |
| Variable | B | β | SE | *t* | F | F Δ | *R*^2^ | *R*^2^ Δ |
| Model 1 |  |  |  |  | 14.332*** | - | .406 | - |
| TIV | .000 | .591 | .000 | 5.201*** |  |  |  |  |
| Sex | .000 | .000 | .016 | .000 |  |  |  |  |
| Age | -.003 | -.170 | .001 | -2.001* |  |  |  |  |
| Placebo | .002 | .188 | .001 | 2.214* |  |  |  |  |
| Model 2 |  |  |  |  | 11.782*** | 1.346 | .415 | .009 |
| TIV | .000 | .588 | .000 | 5.183*** |  |  |  |  |
| Sex | .000 | .002 | .016 | .016 |  |  |  |  |
| Age | -.003 | -.186 | .001 | -2.170* |  |  |  |  |
| Placebo | .002 | .187 | .001 | 2.215* |  |  |  |  |
| Alcohol | .001 | .099 | .001 | 1.160 |  |  |  |  |
| Notes: *p<0.05, **<0.01, ***<0.001 | | | | | | | | |
